# Indocyanine Green Fluorescent Lymphography for Preservation of Spermatic Cord Lymphatic Vessels during Robotic Inguinal Hernia Repair with No Postoperative Symptomatic Seroma: A Case Report

**DOI:** 10.70352/scrj.cr.26-0377

**Published:** 2026-09-17

**Authors:** Fumitoshi Mizutani

**Affiliations:** Department of Surgery, Nagoya Ekisaikai Hospital, Nagoya, Aichi, Japan

**Keywords:** inguinal hernia repair, robot-assisted surgery, transabdominal preperitoneal repair, robotic transabdominal preperitoneal repair, indocyanine green fluorescence, fluorescent lymphography, lymphatic preservation, postoperative seroma, seroma

## Abstract

**INTRODUCTION:**

Postoperative seroma is a relatively common complication of laparoscopic inguinal hernia repair, particularly in patients with large inguinoscrotal hernias. Recently, indocyanine green (ICG) fluorescent lymphography has enabled the visualization of lymphatic vessels within the spermatic cord, and intraoperative lymphatic injury has been suggested as a cause of postoperative clinically significant or symptomatic seroma. However, few studies have demonstrated the clinical significance of lymphatic preservation.

**CASE PRESENTATION:**

A 52-year-old man presented with right inguinal swelling. CT revealed a right indirect inguinal hernia with the small bowel extending into the scrotum. A robotic transabdominal preperitoneal repair was performed. After induction of general anesthesia, 1 mL of ICG (0.25 mg/mL) was injected into the right testicle, and lymphatic vessels were visualized using the Firefly mode of the da Vinci surgical system. Two lymphatic vessels were clearly identified and carefully preserved during precise dissection. The postoperative course was uneventful, and the patient remained asymptomatic. CT on POD 10 revealed a small amount of serous fluid accumulation. On POD 24, ultrasonography showed that this serous fluid collection had resolved.

**CONCLUSIONS:**

ICG fluorescent lymphography enabled intraoperative visualization and preservation of spermatic cord lymphatic vessels during RTAPP. Although a small asymptomatic fluid collection was observed, no clinically significant seroma developed. Further studies are required to determine whether lymphatic preservation reduces postoperative symptomatic seroma.

## Abbreviations

ICG
: indocyanine green
RTAPP
: robotic transabdominal preperitoneal repair

## INTRODUCTION

A postoperative seroma is a common complication of laparoscopic inguinal hernia repair. According to the 16th Nationwide Survey of Endoscopic Surgery in Japan, seroma occurs in 4.1% of cases and is particularly frequent in large inguinoscrotal hernias.^1,2)^ This may cause patient anxiety and difficulty in distinguishing it from recurrence.^3)^

Seromas have traditionally been attributed to dead spaces. However, recent studies using ICG fluorescent lymphography have demonstrated that lymphatic vessels within the spermatic cord can be visualized, suggesting that lymphatic injury may contribute to postoperative seroma.^4)^

These lymphatic vessels typically consist of 2 main trunks running along the testicular arteriovenous structures and adjacent to the hernia sac, but are difficult to identify using conventional white-light imaging.^5)^ Furthermore, intraoperative lymphatic injury has been reported as an independent risk factor for postoperative seroma.^6)^

However, few studies have demonstrated the clinical significance of preserving these lymphatic vessels. Herein, we report a case of an inguinoscrotal hernia in which the lymphatic vessels were preserved using ICG fluorescent lymphography during RTAPP, resulting in a postoperative asymptomatic seroma. To the best of our knowledge, this is the first report describing intraoperative ICG lymphography during RTAPP. This study was conducted in accordance with the SCARE 2025 guidelines.^7)^

## CASE PRESENTATION

A 52-year-old man presented with right inguinal swelling. He had undergone surgery and radiotherapy for tongue cancer 4 years prior to presentation.

He had noticed right inguinal bulging for 1 year. One month before presentation, he developed acute pain due to incarceration, which was manually reduced at a local clinic. The patient was diagnosed with a right inguinal hernia and was referred to our hospital for surgical treatment.

Physical examination revealed a large reducible swelling extending from the right inguinal region to the scrotum. Laboratory findings were unremarkable.

**Fig. 1:**
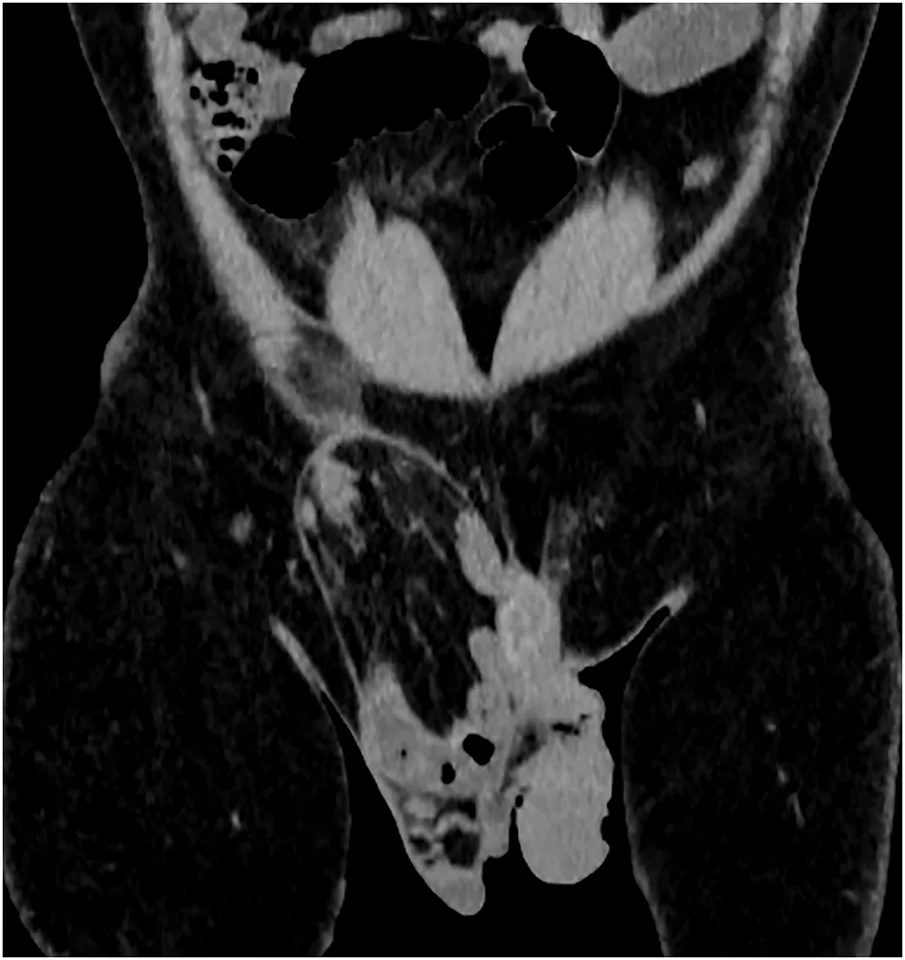
CT revealed a right indirect inguinal hernia with the small bowel prolapsing into the scrotum.

CT revealed a right indirect inguinal hernia with small bowel prolapse into the scrotum (**Fig. 1**).

Based on these findings, RTAPP was planned, and intraoperative ICG fluorescent lymphography was performed.

### Surgical findings

**Fig. 2:**
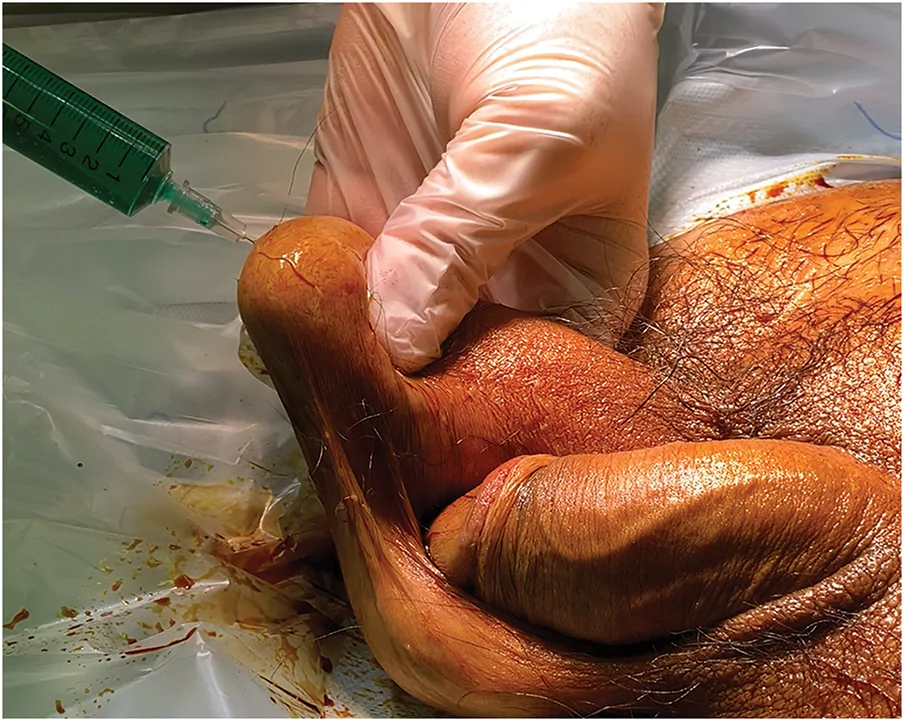
After the induction of general anesthesia, 1 mL of intraoperative ICG (0.25 mg/mL) was injected directly into the right testicle, followed by gentle scrotal massage. ICG, indocyanine green

After the induction of general anesthesia, 1 mL of ICG (0.25 mg/mL) was injected directly into the right testicle, followed by gentle scrotal massage (**Fig. 2**). Fluorescence was detected 61 seconds after intratesticular ICG injection. Because the fluorescence was maintained throughout the procedure, no additional ICG administration was required.

Robotic surgery was performed using three 8-mm ports. A right indirect inguinal hernia with an orifice of approximately 4 cm was identified, and the hernia sac extended into the scrotum.

**Fig. 3:**
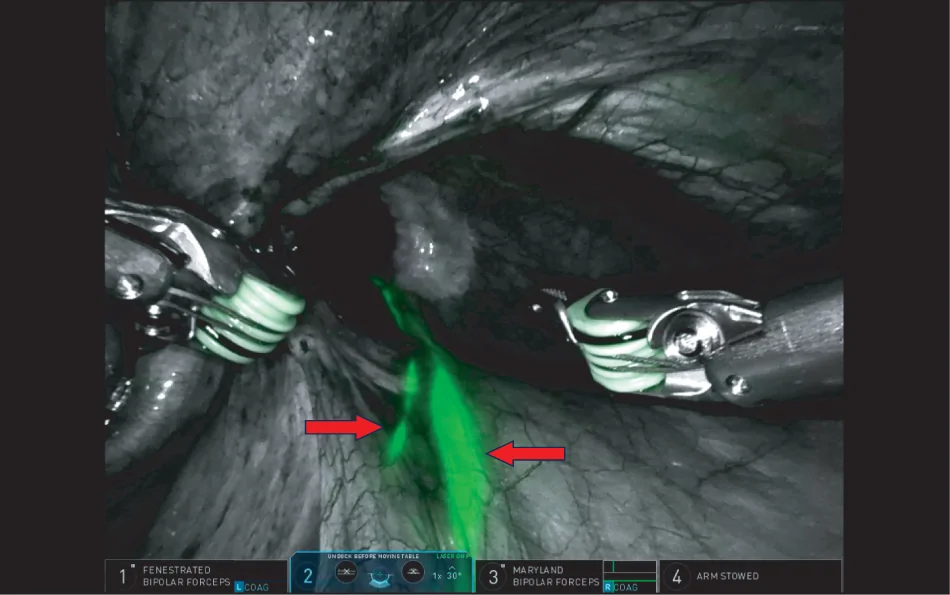
Using the Firefly mode of the da Vinci surgical system, 2 lymphatic vessels running within the spermatic cord were clearly visualized. The 2 lymphatic vessels are indicated by arrows.

Two lymphatic vessels running within the spermatic cord were clearly visualized using the Firefly mode of the da Vinci surgical system (**Fig. 3**). These appeared as linear fluorescent structures that could not be identified using standard white-light imaging.

**Fig. 4:**
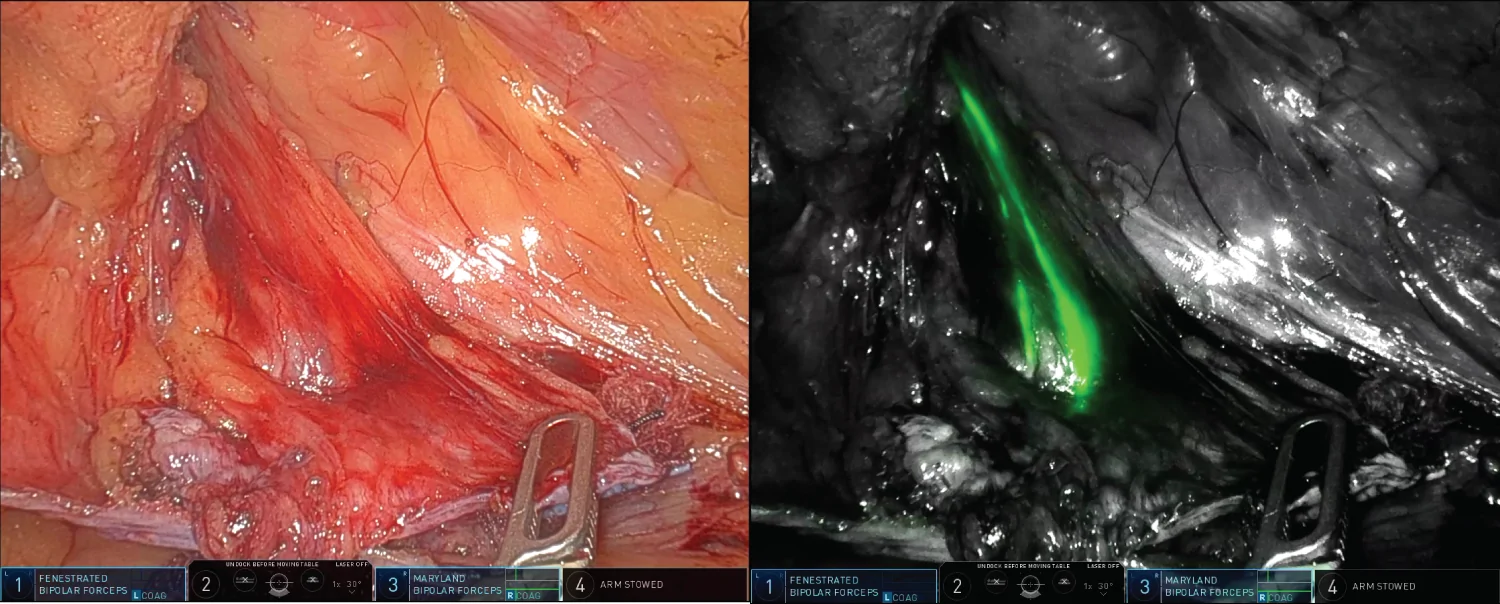
A Firefly mode image obtained during the dissection.

**Fig. 5:**
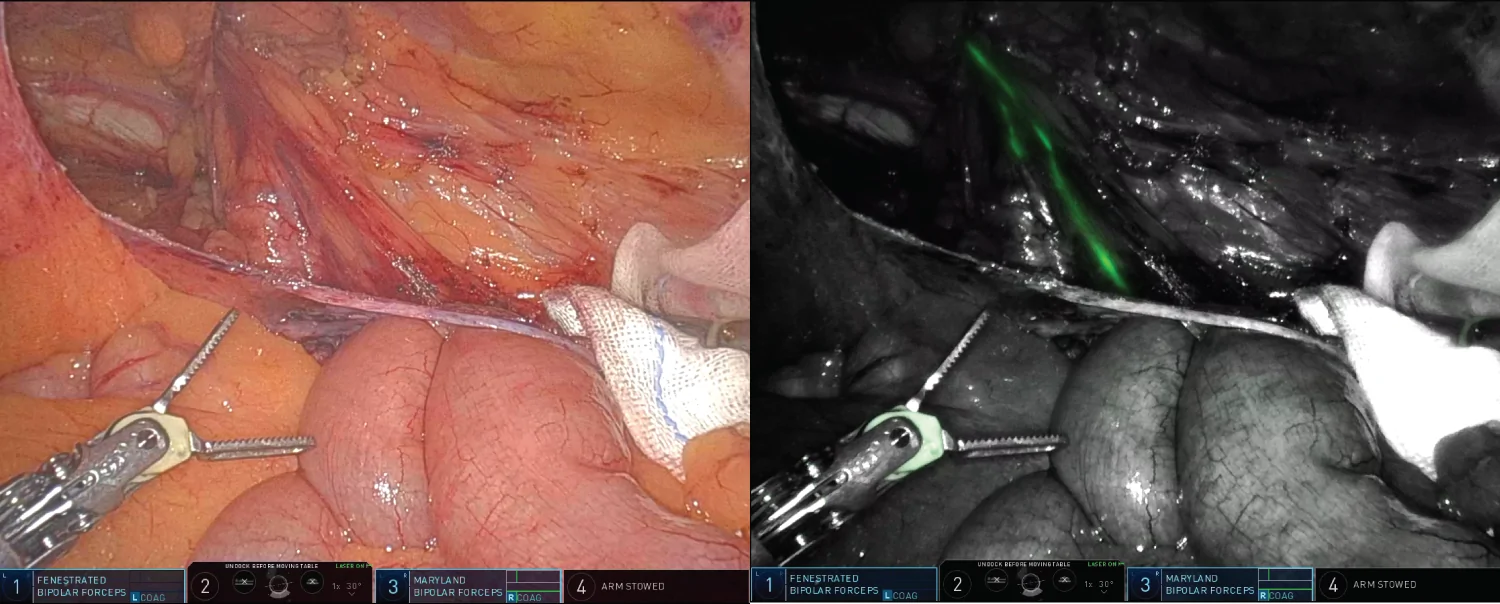
A Firefly mode image obtained after completion of the dissection.

Careful dissection was performed while preserving the lymphatic vessels. The hernia sac was long and circumferentially transected. During parietalization of the spermatic vessels, particular attention was required because of the proximity between the lymphatic vessels and peritoneum (**Fig. 4**); however, safe dissection was achieved (**Fig. 5**).

The lymphatic vessels were preserved completely until the end of the procedure.

A 12 × 16-cm self-gripping mesh was placed, and the peritoneum was closed with continuous sutures.

The operative time was 126 min, and blood loss was minimal (1 g). The patient was discharged on POD 1 without any complications. No adverse events related to ICG treatment were observed.

The postoperative course was uneventful, and the patient remained asymptomatic.

Testicular pain was not observed.

**Fig. 6:**
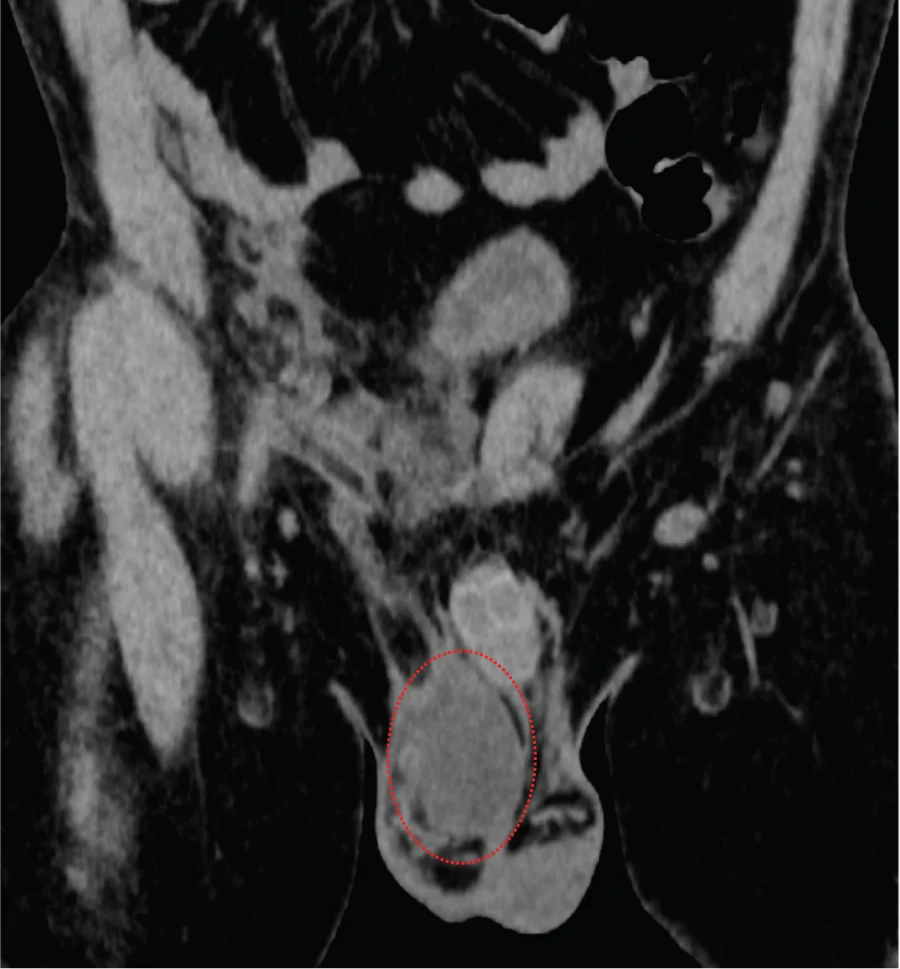
CT on POD 10 revealed a small amount of serous fluid accumulation. A serous fluid collection measuring 2 cm × 2 cm × 3 cm was observed in the right hemiscrotum, as indicated by the red dotted circle.

CT on POD 10 revealed a small amount of serous fluid accumulation. A serous fluid collection measuring 2 cm × 2 cm × 3 cm was observed (**Fig. 6**). On POD 24, ultrasonography showed that this serous fluid collection had resolved.

## DISCUSSION

Postoperative seroma is one of the most common complications of laparoscopic inguinal hernia repair, with reported rates of up to 17.6%,^8)^ especially in large inguinoscrotal hernias.^2)^

Various preventive techniques have been proposed, including drainage,^9)^ closure of the hernia defect,^10)^ and fixation of the transversalis fascia to the Cooper ligament by suturing or tacking.^11,12)^ However, these methods may increase cost, operative time, or postoperative pain.

Studies have histologically confirmed that the fluorescent vessels identified by ICG are lymphatic vessels.^4)^ Furthermore, intraoperative lymphatic injury has been demonstrated to be an independent risk factor for postoperative seroma.^6)^

Based on these observations, postoperative seroma may be classified into 2 types according to its pathophysiology. One type is caused by the accumulation of reactive peritoneal fluid associated with surgical manipulation, while the other results from the addition of lymphatic leakage due to intraoperative lymphatic vessel injury.

The former typically involves a small volume of fluid, is asymptomatic, and resolves spontaneously within a short period. In contrast, the latter is characterized by a larger volume due to persistent lymphatic leakage, tends to increase progressively, is often symptomatic, and requires a longer time to resolve.

In the present case, although a certain amount of reactive peritoneal fluid likely accumulated due to the large inguinoscrotal hernia, lymphatic vessel injury was successfully avoided using ICG fluorescent lymphography. Therefore, no additional lymphatic leakage occurred, which may have avoided a clinically significant or symptomatic seroma. This finding supports the hypothesis that lymphatic preservation plays a key role in reducing clinically significant seroma.

Lymphatic vessels are difficult to identify using white-light imaging and may be unintentionally injured during conventional surgery. ICG fluorescent lymphography enables clear visualization of these structures, allowing lymphatic-preserving surgery.

ICG is safe because it is metabolized by the liver and does not cause discoloration of the urine or scrotum^13)^; however, it should be avoided in patients with iodine allergies.^14)^

Potential concerns associated with direct intratesticular injection of ICG include testicular pain, hematoma, infection, orchitis, vascular injury, possible effects on fertility, and patient acceptability.

Intratesticular injection of ICG is an off-label procedure. At our institution, prior approval for its off-label use was obtained from the Institutional Review Board (IRB). In addition, all patients receive a thorough preoperative explanation of the procedure, and intratesticular ICG injection is performed only after written informed consent has been obtained.

The RTAPP procedure offers additional advantages, including high-definition 3D magnified vision, tremor filtration, and articulated instruments that enable precise dissection. Importantly, the Firefly system allows real-time visualization of lymphatic vessels without the need for additional equipment.

By contrast, conventional laparoscopic TAPP requires a dedicated near-infrared imaging system.^5)^ Thus, RTAPP may facilitate lymphatic preservation.

These findings suggest that RTAPP combined with ICG lymphography is a novel strategy for avoiding clinically significant or symptomatic seromas.

However, ICG fluorescent lymphography itself and intraoperative visualization of lymphatic vessels during inguinal hernia repair have already been reported in both open and laparoscopic surgeries.^4,5)^ Therefore, the novelty of the present case lies in the application of ICG fluorescent lymphography using the Firefly system to RTAPP.

### Limitations

This report describes only a single case and therefore cannot demonstrate that ICG fluorescent lymphography prevents postoperative symptomatic seroma. Rather, it suggests that intraoperative identification and preservation of spermatic cord lymphatic vessels may contribute to the avoidance of clinically significant symptomatic seroma. Larger prospective studies are needed to validate this hypothesis.

## CONCLUSIONS

We report a case in which lymphatic vessels were preserved using ICG fluorescent lymphography during RTAPP, avoiding a clinically significant or symptomatic seroma. Therefore, lymphatic preservation may play an important role in reducing postoperative complications.

## DECLARATIONS

### Availability of data and materials

Data sharing is not applicable to this article as no datasets were generated or analyzed during the current study.
